# Neuromodulation of Gamma-Range Auditory Steady-State Responses: A Scoping Review of Brain Stimulation Studies

**DOI:** 10.3389/fnsys.2020.00041

**Published:** 2020-06-29

**Authors:** Inga Griskova-Bulanova, Kristina Sveistyte, Jovana Bjekic

**Affiliations:** ^1^Institute of Biosciences, Life Sciences Centre, Vilnius University, Vilnius, Lithuania; ^2^School of Life Sciences, University of Nottingham, Nottingham, United Kingdom; ^3^Human Neuroscience Group, Institute for Medical Research, University of Belgrade, Belgrade, Serbia

**Keywords:** auditory steady-state response (ASSR), gamma, brain stimulation, biomarker, neuropsychiatric disorders

## Abstract

Neural oscillations represent a fundamental mechanism that enables coordinated action during normal brain functioning. Auditory steady-state responses (ASSRs) are used to test the ability to generate gamma-range activity. Different non-invasive brain stimulation (NIBS) techniques have the potential to modulate neural activation patterns that are aberrant in a variety of neuropsychiatric disorders. Here, we summarize the current state of knowledge on how different methods of NIBS (transcranial altering current stimulation—tACS, transcranial direct current stimulation—tDCS, transcranial random noise stimulation—tRNS, paired associative stimulation—PAS, repetitive transcranial magnetic stimulation—rTMS) affect the gamma-range ASSRs in both healthy and clinical populations. We show that the current research has been far from systematic and methodologically heterogeneous. Nevertheless, some brain stimulation techniques, especially tACS and rTMS show strong potential for further exploration. We outline the main findings and provide directions for further research into neuromodulation of ASSRs as a promising biomarker of different psychopathological conditions such as schizophrenia, bipolar disorder, attention deficit hyperactivity disorder (ADHD), autism.

## Introduction

Neural oscillations in the gamma band (30–80 Hz) are thought to play a crucial role for information processing in cortical networks (Uhlhaas et al., 2009, 2011; Bosman et al., 2014) as well as are affected in a variety of neuropsychiatric disorders (for review see Herrmann and Demiralp, 2005) and associated with clinical symptoms (McNally and McCarley, 2016; Grent-’t-Jong et al., 2018), cognitive deficits (Bosman et al., 2014) and therapeutic outcomes of pharmaco-treatment (Khalid et al., 2016; Minzenberg et al., 2016; Arikan et al., 2018; Nugent et al., 2019).

Evaluation of brain responses to rhythmically repeated or modulated stimuli allows experimental assessment of gamma activity (Brenner et al., 2009). The largest auditory steady-state responses (ASSRs) recorded with EEG and/or MEG are observed at around 40 Hz (Galambos et al., 1981) with the current views on ASSRs reflecting both the aspect of superposition of middle-latency responses (Presacco et al., 2010) and a periodic activity related to the resonance within the activated system (Santarelli et al., 1995; Ross et al., 2005) These responses, though being distinct from transient evoked gamma (Ross et al., 2005), originate in auditory cortical and subcortical regions (Herdman et al., 2002) with a potential contribution from frontal, motor, parietal and occipital lobes (Farahani et al., 2017, 2019). ASSRs reflect the integrity of neural circuits and excitation/inhibition balance (for details refer to Tada et al., 2019) supported by N-methyl-D-aspartate (NMDA) and γ-aminobutyric acid (GABA) systems (Vohs et al., 2010; Sullivan et al., 2015; Sivarao et al., 2016; Koshiyama et al., 2018b). Accordingly, the alterations of 40-Hz ASSRs were consistently shown in schizophrenia (for review see Thuné et al., 2016) where both preclinical impairment (Tada et al., 2016) and potential to predict treatment outcome (Koshiyama et al., 2018a) were reported, making ASSRs a promising biomarker (O’Donnell et al., 2013). ASSRs were also viewed as indexing for typical and atypical cortical development (Edgar et al., 2016), with variable responses throughout the lifetime (Griskova-Bulanova et al., 2013).

Currently available pharmaco-treatments are relatively ineffective for remediation of cognitive impairments in neuropsychiatric conditions (Goff et al., 2011; Pan et al., 2017; Robbins, 2019). NIBS techniques were proposed as an alternative for pharmaco-treatments and as a cognitive enhancement approach (Cinel et al., 2019; Ishii et al., 2019). The NIBS induces changes in the excitability of target brain areas potentially leading to normalization of altered activation patterns and consequently—to clinically beneficial outcomes (Holtzheimer et al., 2012). Different NIBS techniques use either electrical or magnetic fields to modulate brain activity (for review see Lewis et al., 2016). rTMS uses a magnetic field which is generated by brief current pulses in the coil placed on the scalp, while PAS combines sensory stimulation (e.g., electrical stimulation on the wrist extensor muscle, or auditory stimuli) and magnetic cortical stimulation to induce changes in cortical excitability. Electrical currents (usually up to 2 mA) are used to modulate brain activity between the two electrodes of the opposing polarity in constant (tDCS), altering (tACS) or noise waveform (tRNS) manner. Although the application of these methods has reportedly contributed to the positive outcomes in clinical states (e.g., see Lefaucheur et al., 2020), the effects induced in the brain are not fully understood. According to the growing number of meta-analyses, the efficacy of brain stimulation methods is inconsistent across different conditions, e.g., depression (Razza et al., 2020; Sonmez et al., 2019; Moffa et al., 2020), mild cognitive impairments and dementia (Vacas et al., 2019; Chou et al., 2020; Wang et al., 2020), anxiety and post-traumatic stress (Cirillo et al., 2019), autism spectrum disorders (Barahona-Corrêa et al., 2018), ADHD (Salehinejad et al., 2019), positive and negative symptoms of schizophrenia (Aleman et al., 2018; Kim et al., 2019; Yang et al., 2019). Further research could benefit from the combination of NIBS with different neurophysiological measures like ASSR.

Considering a growing interest in ASSR as a biomarker of neuropsychiatric disorders, it is important to evaluate the effects that NIBS induces on gamma-range ASSRs. Despite its promise for clinical application, this line of research has been pursued sporadically so far. Due to a small number of studies, a comprehensive systematic review is not possible at this time. We attempt to summarize the existing research on the neuromodulation of ASSRs, describe the common patterns found, and define further directions for research on neuromodulation of gamma-range ASSRs.

## Materials and Methods

Online-searches were performed in PubMed and ScienceDirect databases for the keywords “auditory steady-state response,” “auditory steady-state evoked potential,” “auditory entrainment” in combination with “neurostimulation,” “neuromodulation,” “tDCS,” “tACS,” “TES” (transcranial electrical stimulation), “ECT” (electroconvulsive therapy), “TMS.” Potentially relevant articles were also identified by a manual search among the reference lists of included articles. Titles and abstracts were scanned by authors (KS and IG-B) to meet the selection criteria. When the abstract provided insufficient information, the “Materials and Methods” section of the article was reviewed. When a disagreement on the inclusion arose, the last author’s (JB) opinion was sought.

The following inclusion criteria were used: (1) EEG/MEG with gamma-range (30–80 Hz) auditory stimulation; (2) statistical comparison of ASSR measures pre- to post-stimulation; (3) original research articles. Articles published in non-English languages and animal studies were excluded. When articles were not accessible as a full text or lacking necessary information, efforts were made to retrieve missing data by contacting the authors.

From each study, we extracted: (1) sample (type, size, age, gender composition); (2) brain stimulation technique and relevant parameters (electrode/coil position, settings, duration, number of sessions); (3) control group/condition; (4) auditory stimulation settings (stimulation frequencies, stimulation type, number of repetitions, duration); and (5) EEG assessment (site, measures evaluated).

## Results

In total nine articles were included in the review (Table 1, study numbers are further used as references). The majority of studies assessed basic mechanisms and were conducted on young healthy subjects. Only three studies included patient samples—schizophrenia patients were studied in one tACS/tDCS research (St1), and tinnitus patients—in two rTMS studies (St6-7). Overall, both tACS (St1-2, 5) and tDCS (St8-9) were used in three studies each [tDCS acted as a control condition for tACS in one study (St1)]; in addition, one study employed tRNS (St3). The remaining three studies (St4, 6–7) used a variety of TMS-based protocols. The sham condition was used as a control in all except one (St2) of included articles. Additionally, three studies (St1-2, 7) included a different stimulation approach to serve as an active control condition for the specific hypothesis testing. A healthy control group was used only in one (St6) out of three studies in clinical populations. The clinically/behaviorally relevant domains were assessed in four articles, i.e. hallucination prevalence (St1), tinnitus-related impairment (St6-7), and gap detection performance (St2).

**Table 1:** Summary of brain stimulation effects on auditory steady-state responses and methodological descriptions for the included studies.

Nr	Article	Sample size, Type, gender, mean age	Stimulation type/ settings	Control group/condition	Number of sessions	Auditory stimulation	Method to measure ASSR	ASSR results	Behavioral outcome
1	Ahn et al. (2019)	Schizophrenia patients; *N* = 22 (7 f, 15 m), 38.48 ± 10.2 years	**tACS**; Electrodes placement: between F3 and Fp1; between T3 and P3, and Cz (return); Intensity: ± 1 mA (0.04 mA/cm^2^); Frequency: 10 Hz; Duration: 20 min **tDCS**; Electrodes placement: between F3 and Fp1 (anode), between T3 and P3 (cathode); Intensity: ± 2 mA	(1) Sham condition (2) Active control tDCS condition	Two sessions per day for 5 days	Click-trains; 10 Hz, 20 Hz, 30 Hz, 40 Hz and 80 Hz; 90 dB SPL; 200 repetitions each lasting for 500 ms (15 min in total); Binaurally	EEG, 128 electrodes, −0.1 to 0.5 s epoch, amplitude and ITPC	40 Hz ASSR enhancement after tACS; no effect of tDCS	Negative correlation between changes of 40 Hz ASSR and hallucination scores
2	Baltus et al. (2018)	Healthy participants; *N* = 26 (14 f, 12 m), 24 ± 3.2 years	**tACS**; Electrodes placement: Group A at FC5 and TP7/P7, Group B at FC6 and TP8/P8; Intensity: ± 1 mA (0.20 mA/cm^2^); Frequency: ± 4 Hz IGF (median IGF − 49 Hz); Duration: 2 min pre-task (7 min total)	No control. Two Groups: A and B received different stimulation frequency	Single session	1,000 Hz AM, modulated at IGF, at IGF+4 Hz, and IGF − 4 Hz for 10 s twice; intensity not reported; Binaurally	EEG, 32 channel, Fz, Cz, or Pz, amplitude	ASSRs at stimulation frequency increased after tACS	Changes did not resemble gap-detection task performance
3	Van Doren et al. (2014)	Healthy participants; *N* = 14 (7 f, 7 m), 24.6 ± 1.9 years	**tRNS**; Electrodes placement: T6 and T7; Intensity: 2 mA (0.057 mA/cm^2^); Frequency: noise at 101-640 Hz; Duration: 20 min	Sham condition	Single session	1,000 Hz AM; 20 Hz and 40 Hz; 50 dB sensation level; 140 repetitions, each lasting for 800 ms (7 min in total); Binaurally	EEG, 50 electrodes; ROI from F1, Fz, F2, FC1, FCz, FC2, C1, Cz, C2, CP1, CPz, CP2; −2 to 2.5 s epochs; power	40 Hz ASSR enhancement after tRNS	No behavioral assessment
4	Engel et al. (2017)	Healthy participants; *N* = 18 (10 f, 8 m), 21.28 ± 2.37 years	**PAS (TMS)**; Coil placement: 2.5 cm upward from T3 and 1.5 cm in the posterior direction perpendicularly to the line T3–Cz; Settings: 110% MT at 0.1 Hz; 200 auditory stimuli (4,000 Hz) paired with TMS; an interstimulus interval of 45 ms between tone onset and TMS pulse	Sham stimulation	Single session	4,000 Hz AM paired tone/ 1,000 Hz AM control tone; modulated at 20 Hz and 40 Hz; intensity not reported; 200 pair repetitions each lasting for 800 ms; Binaurally	EEG, 64 channel, ROI from F1, Fz, F2, FC1, FCz, FC2, C1, Cz, C2; analyzed 500–800 ms, amplitude	No effect of TMS on 40 Hz ASSR; 20 Hz ASSR decreased after TMS (only with 4,000 Hz AM)	No behavioral assessment
5	Hyvärinen et al. (2018)	Healthy participants; *N* = 18 (6 f, 12 m), 26.6 ± 4.1 years	**tACS**; Electrodes placement: T3 and T4; Intensity: 1.5 mA (0.043 mA/cm^2^); Frequency: 6.5 Hz and 12 Hz; Duration: 5 min	(1) Sham stimulation (2) Two blocks of 6.5 Hz tACS for 1 min	Two sessions	Click-train; 41 Hz; 30 dB above this hearing threshold; Continuous; Binaurally	MEG, 102 magnetometers, 204 planar gradiometers; source power	41 Hz ASSR decreased after 12 Hz tACS; No effect of 6.5 Hz tACS	No behavioral assessment
6	Li et al. (2019)	Tinnitus patients; *N* = 24 (10 f, 14 m), 40-73 years (mean 46) Healthy controls; *N* = 12 (6 f, 6 m), 30-64 years, mean 48)	**rTMS**; Coil placement: BA 41 based on individual MRI; Settings: 110% MT at 1 Hz, 1,800 pulses per session; Duration: 30 min	(1) Healthy controls (2) Sham stimulation in 12 of tinnitus patients	One session per day for 5 days	1,000 Hz AM; 37 Hz; 50 dB SL, 180 s duration twice; Binaurally	MEG, whole-head 306-channel magnetometer, strength of equivalent current dipole	37 Hz ASSR decreased after rTMS course over right hemisphere;	Significant reduction of tinnitus handicap inventory scores
7	Lorenz et al. (2010)	Tinnitus patients; *N* = 10 (3 f, 7 m), 21-70 years (mean 49.8)	**rTMS**; Coil placement: 2.5 cm upward from T3 and 1.5 cm in the posterior direction perpendicularly to the line T3–Cz; Settings: 110% MT; 1) rTMS at 1 Hz 1,000 pulses; 2) rTMS at IAF (8 − 12 Hz, 20 trains with 50 pulses and 25-s intertrain interval); 3) iTBS (10 trains of 10 bursts at a 5 Hz with an 8-s intertrain interval and bursts consisting of three pulses at 50 Hz); 4) cTBS; (at 5 Hz with bursts consisting of three pulses at 50 Hz)	Sham stimulation	One session per stimulation	250 Hz AM, 1,000 Hz AM, 4,000 Hz AM modulated at 40 Hz; set to a loudness of a reference tone (1 kHz, 50 dB SL); 70 repetitions per AM frequency, each lasting for 800 ms; Monaurally to the ear affected by tinnitus	MEG, 148-channel whole-head magnetometer system, strength of ROI around the auditory cortices ipsilateral and contralateral to the TMS stimulation	40 Hz ASSR reduced after iTBS, cTBS and rTMS	Positive correlation between left hemisphere ASSR and tinnitus loudness after rTMS
8	Miyagishi et al. (2018).	Healthy participants; *N* = 24 (all males), 20-23 years (mean 21.3)	**tDCS**; Electrodes placement: F3 (anode) and F4 (cathode); Intensity: 2 mA; Duration: 13 min	Sham condition	Two sessions with 20 mins break	Click-train; 40 Hz; 80 dB SPL; 250 trials, each lasting for 1,000 ms with an inter-trial interval of 2,000 ms; Binaurally	MEG, 160 channel, −500 to 1,500 ms epochs, ERSP and ITPC of ROI	No effect of tDCS on 40 Hz ASSR	No behavioral assessment
9	Pellegrino et al. (2019)	Healthy participants; *N* = 15 (12 f, 3 m), 20-50 years (mean 28.8 ± 3)	**tDCS**; Electrodes placement: C3 (anode) and C4 (cathode); Intensity: 2 mA (0.057 mA/cm^2^); Duration: 20 min	Sham condition	Single session	1,000 Hz AM; 40 Hz; 85 dB; 180 repetitions each lasting 1,000 ms; Binaurally	MEG, 275 gradiometers, −1.5 s to +1.5 s epochs, ITPC and power of individual source	40 Hz less synchronized after tDCS	No behavioral assessment

*Abbreviations: ASSR, auditory steady state response; tACS, transcranial alternating current stimulation; tDCS, transcranial direct current stimulation; tRNS, transcranial random noise stimulation; PAS, paired associative stimulation; rTMS, repetitive transcranial magnetic stimulation; iTBS, intermittent theta burst stimulation; cTBS, continuous theta burst stimulation; IGF, individual gamma frequency; IAF, individual alpha frequency (IAF); AM, amplitude modulated; ITPC, inter-trial phase coherence; ERSP, event-related spectral perturbation; MT, motor threshold; ROI, region of interest*.

Five studies (St5-9) evaluated MEG as an outcome measure. 40-Hz ASSR was assessed in five reports (St1, 3, 4, 7–9); three studies utilized near 40 Hz stimulation (individual gamma frequencies around 50 Hz (St1); 41 (St5) or 37 Hz (St6)); additionally, responses to 20 Hz (St1, 3–4) and 10, 30 and 80 Hz (St1) stimulation were evaluated. Monaural presentation of sounds was used only in two tinnitus-oriented reports (St6-7); remaining studies employed binaural stimulation. The duration of auditory stimuli varied from 180 ms to the continuous sound presentation and only three studies (St1, 5, 8) utilized stimulation with click trains. All included studies focused on either amplitude or power measures of the ASSRs; three studies (St1, 8–9) additionally evaluated phase-locking properties of the response (inter-trial phase coherence, ITPC). Activity at the source level was assessed in three MEG-based works (St5-6, 9); remaining articles reported the measures at the region-of-interest basis. The effects induced by the course of stimulation (5 daily sessions) with tACS (St1) and TMS (St6) were estimated in two studies; single or double daily stimulation was performed in the remaining reports.

Of the reviewed articles, two tACS reports (St1-2) and one tRNS-based report (St3) observed 40-Hz ASSR increase after the stimulation, and one study (St5) reported decreased 41-Hz ASSR after tACS. Among the included studies, two utilized tACS at the alpha frequency (10 Hz (St1) and 12 Hz (St5)); however, both reports produced contradictory results on 40-Hz ASSR. Three studies did not find a tDCS effect on the power of 40-Hz ASSR (St1, 8–9); however, in one study a drop of phase-locking after stimulation was reported (St9). TMS-based protocols resulted in a decrease of ASSRs at 20 (St4), 37 (St6), and 40 Hz (St7).

## Discussion

We assessed available studies addressing the effects of neuromodulatory/brain stimulation approaches on the gamma-range auditory steady-state responses and summarized their results.

Among the most consistent results are two reports on the rTMS-induced reduction of 40-Hz ASSR in tinnitus (Lorenz et al., 2010; Li et al., 2019), where rTMS potentially causes long-term depression of synaptic transmission in the auditory cortex (Li et al., 2019). Lorenz et al. (2010) reported significantly attenuated ASSRs along with tinnitus loudness in patients after both 1 Hz rTMS and continuous theta burst rTMS (Lorenz et al., 2010). In support of this, a recent study of Li et al. (2019) provided evidence for the long-term effect of rTMS on both ASSRs and tinnitus reduction (Li et al., 2019). In healthy participants (Engel et al., 2017), no significant changes were observed for 40-Hz ASSRs after the PAS session where TMS and auditory stimuli were delivered 45 ms apart; however, 20-Hz ASSR was decreased pointing to spike-timing-dependent plasticity as a mechanism of action. Collectively, these findings suggest that rTMS might be potentially used in tinnitus where initial gamma-range ASSRs are elevated; yet, longitudinal studies are needed for more powerful evidence.

Spike-timing-dependent plasticity is proposed as a mode of action of tACS (Tavakoli and Yun, 2017). Although gamma-range tACS has been used to modulate various behavioral aspects in both visual and auditory domains (Rufener et al., 2016; Gonzalez-Perez et al., 2019), surprisingly, no studies published in full to date evaluated the effect of periodic 40 Hz electrical stimulation on the periodic 40 Hz brain responses. In the conference abstract by Knott et al. (2019) (not included in the review due to insufficient information), authors provide initial evidence that no effects of 40 Hz tACS (vs. sham tACS) on 40-Hz ASSRs could be observed (Knott et al., 2019). However, among reviewed studies, Baltus et al. (2018) reported an increase of the gamma-range responses at the stimulation frequencies around 50 Hz compared to individual estimated responses during the rest. Overall, increasing evidence suggests that tACS may modulate cross-frequency interactions (Herrmann et al., 2016). Accordingly, among the reviewed studies, two utilized tACS at lower frequencies—theta (6.5 Hz tACS) and alpha (10 and 12 Hz tACS)—to estimate the role of low-frequency oscillations in the modulation of the 40-Hz ASSRs (Hyvärinen et al., 2018; Ahn et al., 2019). Hyvärinen et al. (2018) suggested that tACS at alpha frequency could induce immediate inhibition of 40-Hz ASSRs. In line with this assumption, the authors observed reduced 40-Hz ASSRs in healthy subjects after a single session of bilateral 12 Hz tACS compared to sham condition. On the contrary, Ahn et al. (2019) expected 10 Hz tACS to enhance 40-Hz ASSRs in schizophrenia patients. The observed augmentation after the course of left-sided stimulation administered for 5 days was linked to the increase in functional connectivity and the reduction in auditory hallucinations. However, a course of five left-sided tACS sessions did not result in evident aftereffects at the 1-week and 1-month follow-up (Ahn et al., 2019), suggesting induced changes being short-lived. Importantly, no significant difference was found in ASSR under the bilateral 6.5 Hz tACS compared to sham in the study by Hyvärinen et al. (2018), and the difference in ASSR change between 12 Hz tACS and 6.5 Hz stimulation conditions was not significant. Nevertheless, in the preliminary report of Knott et al. (2019), bilateral 6 Hz tACS resulted in a significant reduction of amplitude and phase-locking of 40-Hz ASSRs (Knott et al., 2019). In contrast, a single study employing rRNS at 101–640 Hz, resulted in a significant increase of 40-Hz ASSRs when compared to the baseline (Van Doren et al., 2014). These observations suggest the potential for theta, alpha and high-frequency tACS protocols to modulate gamma-range ASSRs. The effect could be due to the direct action on underlying networks (Tavakoli and Yun, 2017) and/or modulation of attention towards auditory stimuli (Hopfinger et al., 2017; Wöstmann et al., 2018), as ASSRs are sensitive to variations in attention level (Skosnik et al., 2007; Griskova-Bulanova et al., 2011). However, the effects of tACS on attention are still unclear, thus this possibility should be addressed in future studies: experimental designs with attentional distraction or audio-visual conflict paradigm may provide insight into top-down vs. bottom-up effects of NIBS on ASSRs.

The effect of tDCS on gamma-range ASSRs, acting through long-term potentiation and long-term depression (LTP/LTD)-like neuroplasticity (Roche et al., 2015), was assessed in three recent studies (Miyagishi et al., 2018; Ahn et al., 2019; Pellegrino et al., 2019). Ahn et al. (2019) employed a left-sided tDCS as a control condition for tACS in schizophrenia patients, expecting no effect of tDCS on 40-Hz ASSRs; on the contrary, Miyagishi et al. (2018) and Pellegrino et al. (2019) anticipated modulation of 40-Hz ASSRs in healthy subjects. Both Ahn et al. (2019) and Miyagishi et al. (2018) did not find changes of ASSR after the stimulation, whereas Pellegrino et al. (2019) reported a decrease in phase-locking of 40-Hz ASSRs in the right temporal cortex without a significant influence on response power (Pellegrino et al., 2019) after a single session of tDCS over the central region. These findings point to the potential site-specific and measure-specific effects of tDCS.

Altogether, it seems that different neuromodulatory techniques can produce physiologically relevant changes in ASSRs. However, this rather new line of research should adopt standardization of brain stimulation protocols (e.g., for TMS see Lefaucheur et al., 2020) to avoid some of the most common issues in NIBS research. First, the use of sham-controlled designs is essential; even though some sham-protocols were shown to produce effects beyond intended sensational masking (for discussion see Duecker and Sack, 2015; Fonteneau et al., 2019; Zis et al., 2020), the success in blinding procedures and any unexpected sham-effects should be reported. Second, the consistency of stimulation protocols applied is needed to increase reproducibility. So far, frontal (Miyagishi et al., 2018; Ahn et al., 2019), sensory-motor (Pellegrino et al., 2019) and temporal (Hyvärinen et al., 2018) areas were stimulated to achieve modulation of ASSRs; however, the primary modulation target for auditory responses should be the auditory cortex. As 40-Hz ASSRs are somewhat asymmetric across the hemispheres, with a right-hemispheric dominance (Ross et al., 2005; Draganova et al., 2008) in healthy subjects, and diminished left-right hemispheric asymmetry in schizophrenia and bipolar disorder (Teale et al., 2003; Reite et al., 2009; Tsuchimoto et al., 2011), the bipolar homolog positioning of the electrodes for TES and TMS coil navigation following MRI-based atlases (Jiang et al., 2020) could be an appropriate starting point. If targeting at temporal lobes is ineffective, other areas beyond auditory pathways should be considered (see Farahani et al., 2017, 2019). Third, sample sizes in the reviewed studies are relatively small (10–26 participants) with no *a* *priori* or *a posteriori* power estimations provided. *A priori* power analysis (e.g., using free tools such as G * Power; Faul et al., 2007) and, where appropriate, adoption of within-subject study design to control the interindividual differences (Li et al., 2015) should help to achieve higher reproducibility. Furthermore, it must be noted that only four studies (Lorenz et al., 2010; Baltus et al., 2018; Ahn et al., 2019; Li et al., 2019) evaluated behavior output alongside EEG/MEG assessment. The majority of neuromodulation studies use either physiological or behavioral outputs; however, the concurrent monitoring of neurophysiological and behavioral performance is necessary to gain a better understanding of NIBS effects (Abellaneda-Pérez et al., 2020). Moreover, even though tACS and TMS show a great promise, it remains unclear which NIBS technique most effectively modulates neural circuits underlying ASSRs; thus, future studies should comparatively assess the effects of multiple brain stimulation methods (Ahn et al., 2019). Finally, only two studies (Ahn et al., 2019; Li et al., 2019) assessed long-term changes after the stimulation course; future research should investigate longitudinal effects on the neural activity, their duration, and potential functional side-effects.

## Conclusion

There is a limited amount of research analyzing the impact of neuromodulation on gamma-range ASSRs. It has been shown that ASSRs can be either enhanced, decreased, or stay unaffected following NIBS with inconsistent findings both within and across different stimulation approaches. The research on rTMS has shown promising results in regards to the reduction of ASSRs in tinnitus; however, future research should aim to provide more evidence on the long-term effects of rTMS. Among TES protocols, tACS at theta, alpha, and high frequencies showed the potential to modulate gamma-range ASSRs. However, future investigations are necessary to explore the mechanisms underlying the modulation of gamma-range ASSRs to develop the non-invasive brain stimulation-based treatments for abnormal auditory processing. A standardization of brain stimulation protocols and ASSR assessment approaches, more rigorous study designs (including adequate control condition, *a priori* power-based determination of sample sizes, minimizing and controlling for potential confounding factors, etc.) as well as inclusion of a wide range of outcome measures to account for unexpected findings, would be beneficial for this research field.

## Author Contributions

IG-B and JB designed the work. KS and IG-B analyzed the data. IG-B and KS drafted the manuscript. JB critically reviewed the manuscript. All authors approved the manuscript.

## Conflict of Interest

The authors declare that the research was conducted in the absence of any commercial or financial relationships that could be construed as a potential conflict of interest.
